# Longitudinal Cerebral Structural, Microstructural, and Functional Alterations After Brain Tumor Surgery for Early Detection of Recurrent Tumors

**DOI:** 10.3390/biomedicines13112811

**Published:** 2025-11-18

**Authors:** Rebecca Kassubek, Mario Amend, Heiko Niessen, Bernd Schmitz, Jens Engelke, Nadja Grübel, Jochen Weishaupt, Karl Georg Haeusler, Jan Kassubek, Hans-Peter Müller

**Affiliations:** 1 Department of Neurology, University Hospital Ulm, 89081 Ulm, Germanyjan.kassubek@uni-ulm.de (J.K.);; 2 Boehringer Ingelheim Pharma GmbH & Co. KG, 88397 Biberach, Germany; 3 Department of Diagnostic and Interventional Radiology, University Hospital Ulm, 89081 Ulm, Germany; 4 Department of Neurosurgery, Bezirkskliniken Guenzburg, 89312 Guenzburg, Germanynadja.gruebel@bkh-guenzburg.de (N.G.); 5 Department of Nuclear Medicine, University Hospital Ulm, 89081 Ulm, Germany

**Keywords:** brain tumor, surgery, diffusion tensor imaging, magnetic resonance imaging, resting state MRI, glioma

## Abstract

**Background**: Early detection of recurrent brain tumors after malignant glioma surgery is a challenge in imaging-based assessment of glioma. **Objective**: The aim of this case series is to investigate whether there are signs for an improvement in the early detection of recurrent tumors using multiparametric magnetic resonance imaging (MRI) after glioma surgery. **Methods**: An MRI protocol was used with high-resolution fluid-attenuated inversion recovery (FLAIR), diffusion tensor imaging (DTI), resting state functional MRI (rsfMRI), and contrast-enhanced high resolution T1-weighted (T1w). Longitudinal multiparametric MRI was performed in six patients with glioblastoma with one complete scan before surgery, one scan after surgery and at least two follow-up scans. A total of 27 complete multiparametric MRI data sets were available. **Results**: DTI analysis at the localizations of recurrent tumors showed early directionality loss in DTI by fractional anisotropy (FA) reduction accompanied by FLAIR hyperintensities before hyperintensities in contrast enhanced T1w were visible. One out of six patients showed a regional FA decrease at the localization of the recurrent tumor at a point of time even when the morphological T1w- and FLAIR images did not demonstrate any detectable changes. Functional connectivity alterations in a corresponding network could also be detected at the localizations of the recurrent tumor. **Conclusions**: In addition to routine T2w FLAIR and contrast enhanced T1w, DTI and rsfMRI might complement information for the early detection of recurrent malignant glioma. Prospective studies at larger scale are needed with respect to potential of DTI and rsfMRI for early recurrent tumor detection.

## 1. Introduction

Glioma are a heterogeneous group of brain tumors that are diagnosed according to their histological tissue features and their distinct molecular biomarkers [1], as summarized in the 2021 WHO classification of central nervous system tumors [2]. Especially the most common grade 4 CNS tumors, glioblastoma, are highly aggressive: despite multidisciplinary treatment approaches with a combination of radiotherapy and chemotherapy (with the alkylating agent temozolomide), the prognosis for high-grade glioma is still poor, and the relative survival rate estimates are 43% after one year and 7% after 5 years [1]. As with systemic malignancies, great efforts are being made in neuro-oncology to find new therapeutic strategies to improve patients’ lifespan and quality of life. Neuroimaging with a focus on magnetic resonance imaging (MRI) has been used to characterize tumors, to define malignant and active areas, to assess disease prognosis, and to quantify and monitor therapy response [3,4]. Malignant gliomas usually recur even after complete resection followed by radiotherapy and chemotherapy, so that other therapeutic approaches such as targeted, individualized therapies are currently being developed and investigated [1]. Longitudinal imaging by MRI has been used to investigate GBM patients during therapy and to identify factors like rapid early progression for prognostic reasons [5] or to perform survival risk stratification [6]. Some of these novel therapeutic approaches are hard to be adequately monitored by current standard imaging techniques. Novel machine-learning (ML)-based applications to brain MRI can substantially add to the imaging-based detection of brain tumors [7]. In addition, new imaging findings underscore the role of structural and functional connections within hierarchical networks of the connectome [8]. However, the effect of these therapeutic approaches in terms of structural and functional changes in the brain remains to be further explored in a clinical setting. It has been discussed that MRI techniques may be useful as a biological marker [9,10] in monitoring the distinct effects of the emerging new therapeutic concepts on brain integrity, as they might lead to changes in structural and functional connectivity due to various changes in vasculature and microenvironment which can be assessed by techniques like diffusion tensor microstructure imaging (DTI) for structural and intrinsic functional connectivity (ifc) MRI for functional connectivity imaging.

The following MRI techniques are subjected for application to glioma patients: T1-weighted (T1w) and T2-weighted (T2w)/fluid attenuated inversion recovery (FLAIR) morphological imaging [9,11,12], DTI [13,14,15] and other diffusion weighted imaging (DWI) [16] and ifc MRI [17,18]. Combinations of these approaches can be used to investigate glioma in more detail including DTI [19] and ifcMRI [20], which was the rationale for the current clinical case series report which aims to evaluate the value of multiparametric MRI for the detection of recurrent brain tumors after glioma surgery by the combination of DTI and rsfMRI in addition to high-resolution FLAIR and contrast-enhanced high resolution T1w. The aim of this clinical case series was to examine longitudinal structural, microstructural, and functional alterations both in the ipsilateral as well as in the contralateral hemisphere in association with high-grade glioma to investigate the potential clinical use of these imaging parameters as (non-invasive) biological markers for follow-up monitoring.

## 2. Methods

### 2.1. Subjects and Inclusion Criteria

The subjects’ characterization and the scan statistics are summarized in Table 1. Six patients (four males and two females, mean age 62 years, range 41 to 76) with glioblastoma were included in this prospective study. All patients had the histologically confirmed diagnosis of glioblastoma, IDH wildtype. Four patients had undergone complete tumor resection in initial surgery; two patients had undergone near complete resection. All subjects gave written informed consent for study inclusion in accordance with the Declaration of Helsinki. The local ethics committee of the University of Ulm had approved the study (reference #493/21).

### 2.2. MRI Scanning Protocols

All MRI data were recorded on the same 3.0T Siemens Magnetom Vida (Siemens Medical, Erlangen, Germany) with a 12-channel head coil.

#### 2.2.1. T1w

Contrast-enhanced T1w data included 192 axial slices (1.0 mm thickness), recorded with a 256 × 192 matrix, interpolated to 0.47 × 0.47 mm^2^ in-plane resolution in a 512 × 496 matrix, echo time (TE) = 3.2 ms, and repetition time (TR) = 1530 ms; total acquisition time (TAT) = 5 min.

#### 2.2.2. T2w FLAIR

T2w FLAIR data included 160 axial slices (1.1 mm thickness), recorded with a 256 × 192 matrix, interpolated to 0.35 × 0.35 mm^2^ in-plane resolution in a 512 × 512 matrix, TE = 340 ms, and TR = 5000 ms, TI = 1800 ms; TAT = 13 min.

#### 2.2.3. DTI

DTI data were acquired using 65 gradient directions (GD), 1 GD with b = 0, and 64 GD with b = 1000 s/mm^2^, TE = 81 ms, TR = 5200 ms; 72 axial slices, in-plane matrix 114 × 114, voxel size 2.0 × 2.0 × 2.0 mm^3^; TAT = 6 min.

#### 2.2.4. rsfMRI

RsfMRI data were acquired using 200 volumes, TE = 30 ms, TR = 1460 ms; 48 axial slices (3.3 mm thickness), in-plane matrix 94 × 94, in-plane resolution 2.3 × 2.3 mm^2^; TAT = 5 min.

Longitudinal scans were available for all six patients; one complete scan before surgery (within a maximum of 1 week), one scan within 2 days after surgery and at least two follow-up scans every 3 months. In summary, 27 complete multiparametric MRIs were available (Table 1). The whole MRI data presentation protocol including dropouts are summarized in Supplementary Figure S7. Dropout rate was 9/15. Six patients obtained baseline (pre/post surgery) and complete MRI (T1w, T2w, DTI, ifcMRI) at a minimum of two follow-up visits.

### 2.3. Data Analysis

The DTI analysis software *Tensor Imaging and Fiber Tracking* (TIFT) (version 2025) [14,21] was used for the data processing.

#### 2.3.1. Preprocessing: Alignment of Individual Longitudinal Scans and Parameterizations

In order to avoid bias from the baseline scan, both the baseline and the longitudinal T1w, T2w, DTI-based b0 maps (recordings with no diffusion encoding gradients), and first volumes of rsfMRI scans were aligned to the AC-PC line by a rigid brain transformation. DTI and rsfMRI scans were motion corrected, DTI scans were corrected for eddy currents. All data were controlled for additional artifacts [14].

T1w and T2w scans were used for visual detection of intensity abnormalities by an experienced neuroradiologist (BS). For the spatially aligned scans, FA maps were calculated—for details of DTI data analysis, refer to [22,23]. RsfMRI data underwent a standard preprocessing protocol [24], that was prepared to identify seed-based intrinsic functional connectivity networks.

#### 2.3.2. Post-Processing: Region of Interest (ROI) Analyses

**Figure 1:**
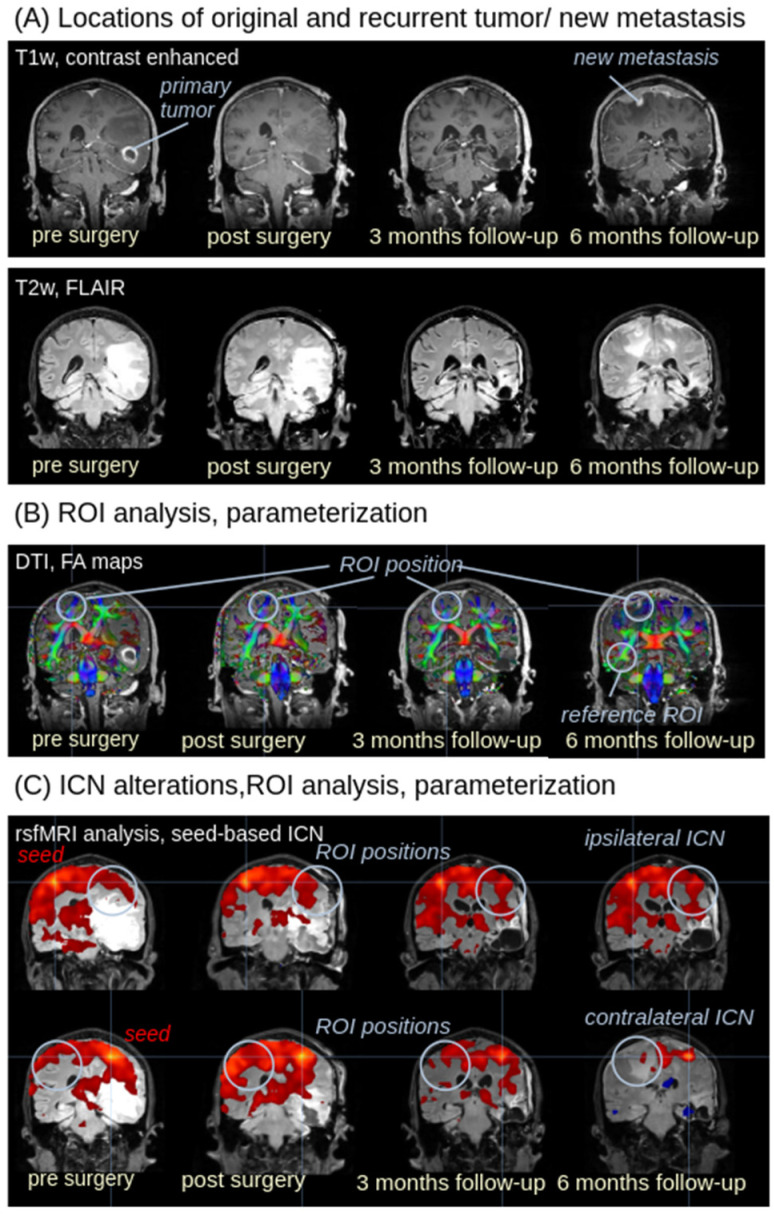
Analysis strategy for multiparametric MRI. The analysis cascade is demonstrated in images of subject 001 (male, age 69 years). (**A**) The postsurgical lesion was identified in T1w and T2w FLAIR images. In follow-up scans, the recurrent tumor could be identified in T1w and T2w FLAIR images as well. (**B**) Region of interest (ROI) (light blue) in FA maps (displayed on a T1w background for visualization purposes). ROIs were positioned at the localization of the recurrent tumor; as a reference, an additional ROI was placed. (**C**) rsfMRI data enable the calculation of intrinsic functional connectivity networks (ICNs) by seed-based analysis with seed localizations at the ipsi- and contralateral site of the recurrent tumor (displayed on a T2w background for visualization purposes); alterations of functional connectivity at the site of the new metastasis (and the same localization at the contralateral hemisphere for comparison, respectively) were calculated by ROI analysis with diameter-extended ROIs (light blue). Upper row: seed for ICN analysis is placed in the left (contralateral) hemisphere (marked by “seed”); lower row: seed for ICN analysisis placed in the right (tumor) hemisphere.

Figure 1 illustrates the analysis strategy for multiparametric MRI analysis of the recurrent tumor. The postsurgical lesion was identified in T1w and T2w FLAIR images (Figure 1A). In DTI-based FA maps, spherical 3-D regions of interest (ROIs) were positioned at the localization of the recurrent tumor and averaged FA values in the respective ROI were calculated for all longitudinal scans. Average FA values were calculated in these ROIs using an FA threshold of 0.2, since grey matter and tumor tissue show FA values < 0.2 [25]; as a reference, an additional ROI was placed in the contralateral hemisphere at the same coordinates (Figure 1B). For rsfMRI data, intrinsic functional connectivity networks (ICNs) were calculated by seed-based analysis with seed localizations at the ipsi- and corresponding contralateral localization of the recurrent tumor (Figure 1C). The ROI-based analyses of functional connectivity in the ICNs were carried out in the contralateral hemisphere of the seed-based analysis. The diameter-extended ROIs were defined to cover a large region of the respective ICN (in the contralateral hemisphere of the ifc seed; the size and position of the ROI is chosen to be fully located in one hemisphere and not to be influenced by the postsurgical lesion.

## 3. Results

### 3.1. Recurrent Tumor in T1w and T2w Images

**Figure 2:**
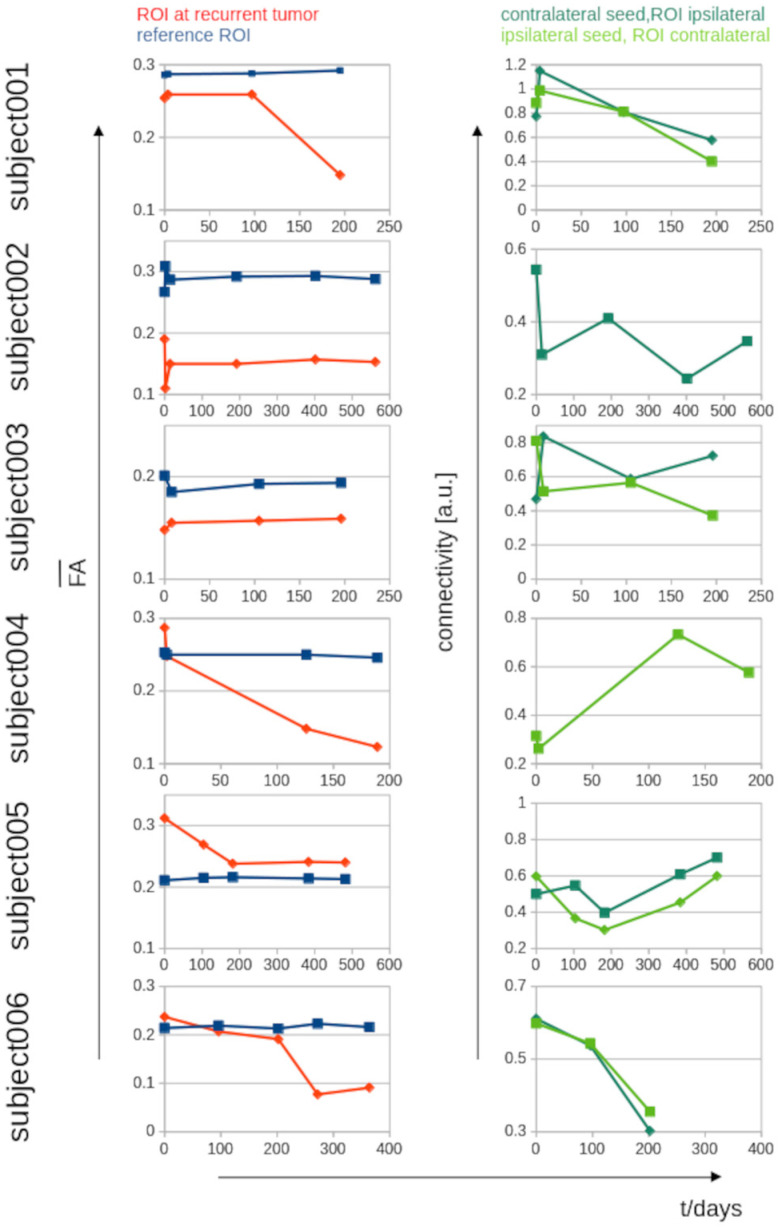
Parameterized longitudinal FA alterations and longitudinal ICN alterations for the six subjects over time. Left panel: Averaged FA values in spherical 3-D ROIs at the localization of the recurrent tumor and at the reference region for all longitudinal scans. Right panel: Intrinsic functional connectivity in ROIs at the network localization of the recurrent tumor and the corresponding network localization in the contralateral hemisphere.

Supplementary Figures S1–S6 show representative images of the multiparametric MRI (with a TAT of 30 min for the four sequences analyzed in this study) of postsurgical lesion and recurrent tumor in T1w and T2w images as well as ROI localizations in DTI-based FA maps and rsfMRI-based ICNs. Subjects 001, 004, and 006 showed a recurrent tumor at localizations distant from the postsurgical lesion, whereas the subjects 002, 003, and 005 showed a recurrent tumor adjacent to the postsurgical lesion. Figure 2 shows the images of a recurrent tumor depending on the time of scan (detected by hyperintensity).

### 3.2. ROI Analysis in FA Maps

**Figure 3:**
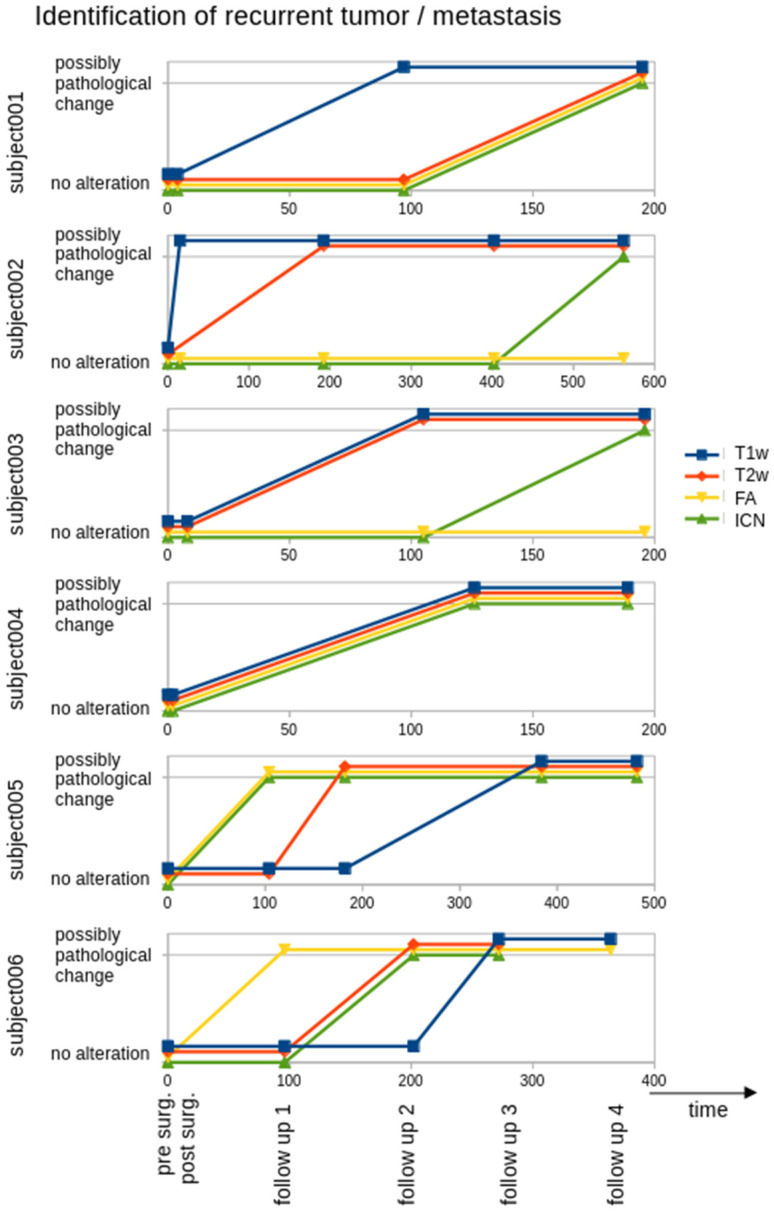
Patient flowchart with appearance of a recurrent tumor/new metastasis (possibly pathological change in a modality—T1w, T2w, FA, ICN) depending on the time of scan. Postsurgical lesion and new metastasis were identified by alterations in T1w and T2w images, recurrent tumor was associated with FA decrease in ROI analysis and/or by alterations of ICN connectivity.

The localization of the recurrent tumor identified in later T2w follow-up scans was defined as the seed for the ROI analysis of FA maps. As a reference, an ROI seed at a different brain localization (where no tumor association could be assumed—Supplementary Figures S1–S6) was defined. Figure 2 shows a summary of FA alterations in the respective ROIs: subjects 001, 004, 005, and 006 showed a decrease in FA at the localization of the recurrent tumor, whereas FA remained constant in the reference ROI. Subjects 002 and 003 showed no decrease in FA at the localization of the recurrent tumor (Supplementary Figures S2 and S3). Figure 3 shows a schematic synopsis of the assessment of recurrent tumor-associated MRI alterations in the patients, displayed as a possibly pathological change for each modality (T1w, T2w, FA, ICN) in time.

### 3.3. ROI Analysis in ICNs

ICNs were identified by seed-based rsfMRI analysis with seeds localized at the site of the recurrent tumor as well as at the corresponding area in the contralateral hemisphere. ICN alterations were hypothesized to appear at the contralateral extension of the ICN (not at or nearby the seed). The ROI analysis (at the contralateral site of the seed-based identified ICN) showed an ICN decrease for subjects 001, 002 (only the contralateral seed could be analysed due to technical reasons), 003, and 006, whereas subjects 004 and 005 showed an ICN increase (Figure 2 and Figures S1–S6). Figure 3 shows a possibly pathological change in ICN in time vs. the further modalities (T1w, T2w, FA).

## 4. Discussion

### 4.1. Multiparametric MRI in the Assessment of (Recurrent) Tumors

This neuroimaging clinical case series report aimed to investigate the potential of multiparametric MRI for early detection of recurrent tumor tissue after glioma surgery and how the associated alterations might progress over time. The longitudinal multiparametric MRI observations demonstrated early directionality loss or alterations in ICNs, respectively, in some patients at the localizations of the recurrent tumor, but these findings cannot lead to diagnostic performance claims yet. However, the ex post facto positioning of ROIs served to determine whether a change in FA or ICNs is detectable at all. For (future) prospective investigations, further unbiased (longitudinal) techniques must be used, such as unbiased voxelwise whole brain-based spatial statistics (WBSS [13]) for FA or the examination of multiple networks [24], respectively.

In the current study, one out of six patients (subject 005) showed a regional FA decrease at the localization of the recurrent tumor at a point in time when the morphological T1w- and FLAIR images did not demonstrate any detectable changes. Such early imaging signs of microstructural alterations might assist in identifying localizations where a recurrent tumor will develop. However, it will need larger numbers of patients and closer monitoring to investigate the potential of this approach.

DTI as an application of DWI [13,16], which is sensitive to the random Brownian motion of water molecules uses parameters like FA, representing the directionality of water diffusion, the mean diffusivity, the radial diffusivity, and the axial diffusivity [26]. DTI can improve the detection of brain metastatic lesions compared to T1w and T2w images and is similar to post-contrast T1w [15]. That way, DTI has already been shown to be a non-invasive tool to probe white matter integrity, identifying white matter microstructural changes at a higher sensitivity than T1w and T2w MRI [27]. There is some evidence that DTI may help in distinguishing between high-grade glioma and brain metastases by the analysis of peritumoral FA values [28] or may help in differentiating tumor progression from pseudoprogression [29]. In addition, DTI is a possible tool to improve radiation therapy target delineation as it has been suggested that DTI detects white matter changes due to tumor tissue itself earlier than standard MRI [30].

Glioma and recurrent glioma identification rely heavily on MRI techniques, and the characteristic presentations by hyperintensity/contrast enhancement in T1w CE and hyperintensity in T2w FLAIR have continuously been improved, currently using ML models and new mathematical frameworks [31,32]. In contrast, the additional contribution of the assessment of structural integrity and microstructural myelin loss (detected by e.g., FA decrease), which are expected in the early stages of glioma, have hardly been studied so far. ICN alterations could result from a loss of functional connectivity or could result from compensatory effects [33]; that way, there is yet limited knowledge about to what extent a recurrent tumor causes alterations in ICNs.

The impact of tumor burden on whole brain rsfMRI provides prognostic information in glioblastoma patients and allows to noninvasively study the effects of glioblastoma on the whole brain [18]. Systemic infiltration of the brain by tumor cells is a hallmark of glioma pathogenesis which may cause disturbances in functional connectivity, that way suggesting the applicability of individual rsfMRI in glioma patients; the analysis of the functional connectome revealed that abnormalities of functional connectivity could be detected not only adjacent to the visible lesion but also in distant brain tissue [34].

Glioblastoma can induce widely distributed changes in whole-brain spectral change in resting state fMRI [18], and functional MRI showed that functional connectivity is affected by glioma throughout the whole brain, even in the nonlesional hemisphere [8,34]. The current clinical case series report in six patients showed that ICN alterations in a respective network could be detected at the localizations of the recurrent tumor. However, in this clinical case series report, we observed a reduction in the specific ICN (in line with Maesawa and co-workers [17]), and also an expansion of the specific ICN could be observed in some patients; this finding might result from the seed-based technique shift of the seed-point due to tumor-induced brain deformation or from compensatory effects. However, due to the low subject numbers the reason for these alterations remains speculative.

### 4.2. Limitations

As with all studies that investigate aspects of high-grade glioma behaviour, there are limitations to this case series which should be highlighted. First, the limited number of patients has to be addressed. Thus, within this clinical case series report, an evaluation of the general potential of multiparametric MRI in detection of recurrent glioma cannot be provided. Rather, the study examined whether there are indications of a possible improvement in detection capabilities; an evaluation can only be carried out in larger-scale studies. Due to the high dropout rate, no statistical validation of the indications of parameter alterations could be provided; the current case series can only illustrate the technical feasibility in the severely ill patients and provide the indications to initiate larger-scale studies at all. Given the longitudinal acquisitions of advanced MRI protocols with an acquisition time of up to 30 min in severely ill patients, 27 complete data sets in the six patients are of clinical value. The high dropout rate might introduce bias to the data analysis with respect to patient characteristics/disease severity; however, a substantial dropout rate has to be accepted in a longitudinal diagnostic study in patients with malignant glioma. In this clinical case series report, the tumor localizations and the extent of resection showed high inter-patient variability (Supplementary Figures S1–S6), which will introduce confounding effects. Higher subject numbers with similar tumor localizations (and also full tumor resections) are needed to confirm the results of this clinical case series report, given that our patient sample was also heterogeneous with respect to both the tumor site and the previous neurosurgical intervention (although this heterogeneity should not bias our results, since FA with a threshold of 0.2 is not influenced by the extent of the resection and remaining tumor mass). The effect of a recurrent tumor on ICNs remains an open issue, thus the interpretation of the alterations as increased or decreased connectivity, respectively, in the ICNs should be a topic of specific future research. Clustering analysis technique can be applied to analyze fMRI time-series data in the context of malignant glioma with great potential for improving the differentiation between various subtypes, which is pivotal for developing personalized therapeutic strategies; ongoing research explores the integration of clustering with deep learning approaches, promising more accurate and efficient segmentation in the future [35]. Finally, this DTI-based study was performed with a clinical scanner at 3.0T and, therefore, further optimized MRI protocols that are available at 7.0T [28] might improve the results. In consequence, this study has to deal with an overall signal-to-noise-ratio which is lower compared to optimized high-field MRI tumor studies. However, given the limited availability of scanners beyond 3.0T, this should even be seen as a strength of the study design, since this study might be considered to be a pilot for future clinical tumor studies that could be performed in a conventional clinical set-up. Compared with 1.5T, imaging by 3.0T MRI showed higher signal-to-noise ratio and contrast-to-noise ratio, which allowed better resolution of smaller focal lesions [36]. It has been suggested that DTI at ultra-high field strengths was possible with improved performance in selected ROIs [37].

### 4.3. Summary and Perspectives

This clinical case series report investigated the potential clinical feasibility and possibilities of multiparametric MRI for the early detection of recurrent tumors. It was demonstrated that DTI and rsfMRI as components of multiparametric MRI could be advantageous in this field. Given that one patient showed FA decrease at the localization of the recurrent tumor before any detectable changes in the clinical routine images, microstructural alterations need to be further studied with respect to their potential of early recurrent tumor detection.

The findings of the current study have to be regarded as preliminary in order to pave the way to perform a larger scale future case series with the intention of enabling us to draw robust conclusions; as requirements we suggest: (i) in the set-up, more specific inclusion/exclusion criteria have to be defined and the follow-up intervals have to be standardized (e.g., 3 months); furthermore, matched healthy controls and patients with stable disease have to be included as comparison groups. (ii) The analysis cascade should contain standardized preprocessing pipelines with motion correction and artifact detection; furthermore, the analysis has to be extended to an unbiased whole-brain analysis. (iii) A statistical work-up should be established with statistical thresholds for clinically meaningful changes in FA and functional connectivity; then, longitudinal mixed-effects models have to be applied to quantify temporal changes in imaging metrics, supplemented by confidence intervals, effect size estimates, sample size calculations, and receiver operating characteristic analysis to evaluate the diagnostic performance.

In order to act as a diagnostic tool, FA analysis and ICN analysis must be developed further, beyond seed-based techniques, to techniques that detect FA or ICN alterations in an unbiased whole-brain analysis, e.g., by artificial intelligence-based techniques [32]. Further research, involving larger patient cohorts and longer follow-up, is necessary to refine therapeutic strategies and improve overall patient outcomes [38]. Nevertheless, due to the high dropout rate, this clinical case series could only provide preliminary data (without statistical inference) that DTI and rsfMRI might complement information in addition to routine T2w FLAIR and contrast enhanced T1w for the early detection of recurrent tumor. Given that one patient showed FA decrease at the localization of the recurrent tumor before any detectable changes in the clinical routine images, microstructural alterations might to be prospectively studied in systematic investigations with respect to their potential of early recurrent tumor detection. The findings of the current study have to be regarded as preliminary in order to pave the way to perform a future study with the intention of enabling us to draw robust conclusions. That way, future MRI acquisition protocols should therefore contain also DTI and rsfMRI to allow the implementation of a pilot study with statistical validation.

## Figures and Tables

**Table 1:** Clinical data of the patients including scan statistics.

Patient (Age/Sex)	Scans	Site of Tumor	Site of Tumor
69/m	4	right temporal lobe	right temporal lobe
41/f	5	right temporal lobe	right temporal lobe
65/m	4	right temporal lobe	right temporal lobe
57/f	4	left frontal lobe	left frontal lobe
76/m	5	right parietal lobe	right parietal lobe
61/m	5	right parietal lobe	right parietal lobe

## Data Availability

The dataset used and analyzed during the current study will be made available by the corresponding author upon reasonable request to qualified researchers.

## Supplementary Materials

The following supporting information can be downloaded at: https://www.mdpi.com/article/10.3390/biomedicines13112811/s1. Supplementary Figure S1: Multiparametric MRI analysis for subject 001. Panels from left to right: T1w-contrast enhanced (CE), T2w-FLAIR, FA maps, ICN from rsfMRI. Parameterized longitudinal FA alterations and longitudinal ICN alterations were displayed over time. Supplementary Figure S2: Multiparametric MRI analysis for subject 002. Panels from left to right: T1w-contrast enhanced (CE), T2w-FLAIR, FA maps, ICN from rsfMRI. Parameterized longitudinal FA alterations and longitudinal ICN alterations were displayed over time. Supplementary Figure S3: Multiparametric MRI analysis for subject 003. Panels from left to right: T1w-contrast enhanced (CE), T2w-FLAIR, FA maps, ICN from rsfMRI. Parameterized longitudinal FA alterations and longitudinal ICN alterations were displayed over time. Supplementary Figure S4: Multiparametric MRI analysis for subject 004. Panels from left to right: T1w-contrast enhanced (CE), T2w-FLAIR, FA maps, ICN from rsfMRI. Parameterized longitudinal FA alterations and longitudinal ICN alterations were displayed over time. Supplementary Figure S5: Multiparametric MRI analysis for subject 005. Panels from left to right: T1w-contrast enhanced (CE), T2w-FLAIR, FA maps, ICN from rsfMRI. Parameterized longitudinal FA alterations and longitudinal ICN alterations were displayed over time. Supplementary Figure S6: Multiparametric MRI analysis for subject 006. Panels from left to right: T1w-contrast enhanced (CE), T2w-FLAIR, FA maps, ICN from rsfMRI. Parameterized longitudinal FA alterations and longitudinal ICN alterations were displayed over time. Supplementary Figure S7: Patient’s MRI visits in days from first MRI scan (before surgery—t = 0). Dropout rate was 9/15. Six patients obtained baseline (pre/post-surgery) and complete MRI (T1w, T2w, DTI, ifcMRI) at a minimum of two follow-up visits.

## Abbreviations

The following abbreviations are used in this manuscript:

DTI/DWI	diffusion tensor imaging/diffusion weighted imaging
FA	fractional anisotropy
FLAIR	fluid attenuated inversion recovery
GD	gradient directions
ifc	intrinsic functional connectivity
ICN	intrinsic functional connectivity network
ML	machine-learning
MRI	magnetic resonance imaging
rsfMRI	Resting state functional
ROI	region of interes
T1w/T2w	T1-weighted/T2-weighted
TE/TR	echo time/repetition time
TAT	total acquisition time
TIFT	Tensor Imaging and Fiber Tracking
