# Supplementary material for: Longitudinal Cerebral Structural, Microstructural, and Functional Alterations After Brain Tumor Surgery for Early Detection of Recurrent Tumors
Source: Biomedicines. 2025 Nov 18;13(11):2811. doi: 10.3390/biomedicines13112811 (PMC12650357; doi:10.3390/biomedicines13112811)
Supplement: Supplementary file 1 [file biomedicines-13-02811-s001.zip › biomedicines-3894902-supplementary.pdf]

Supplementary Figure 1: Multiparametric MRI analysis for subject 001. Panels from left to right: T1w-contrast enhanced (CE), T2w-FLAIR, FA maps, ICN from rsfMRI. Parameterized longitudinal FA alterations and longitudinal ICN alterations were displayed over time.

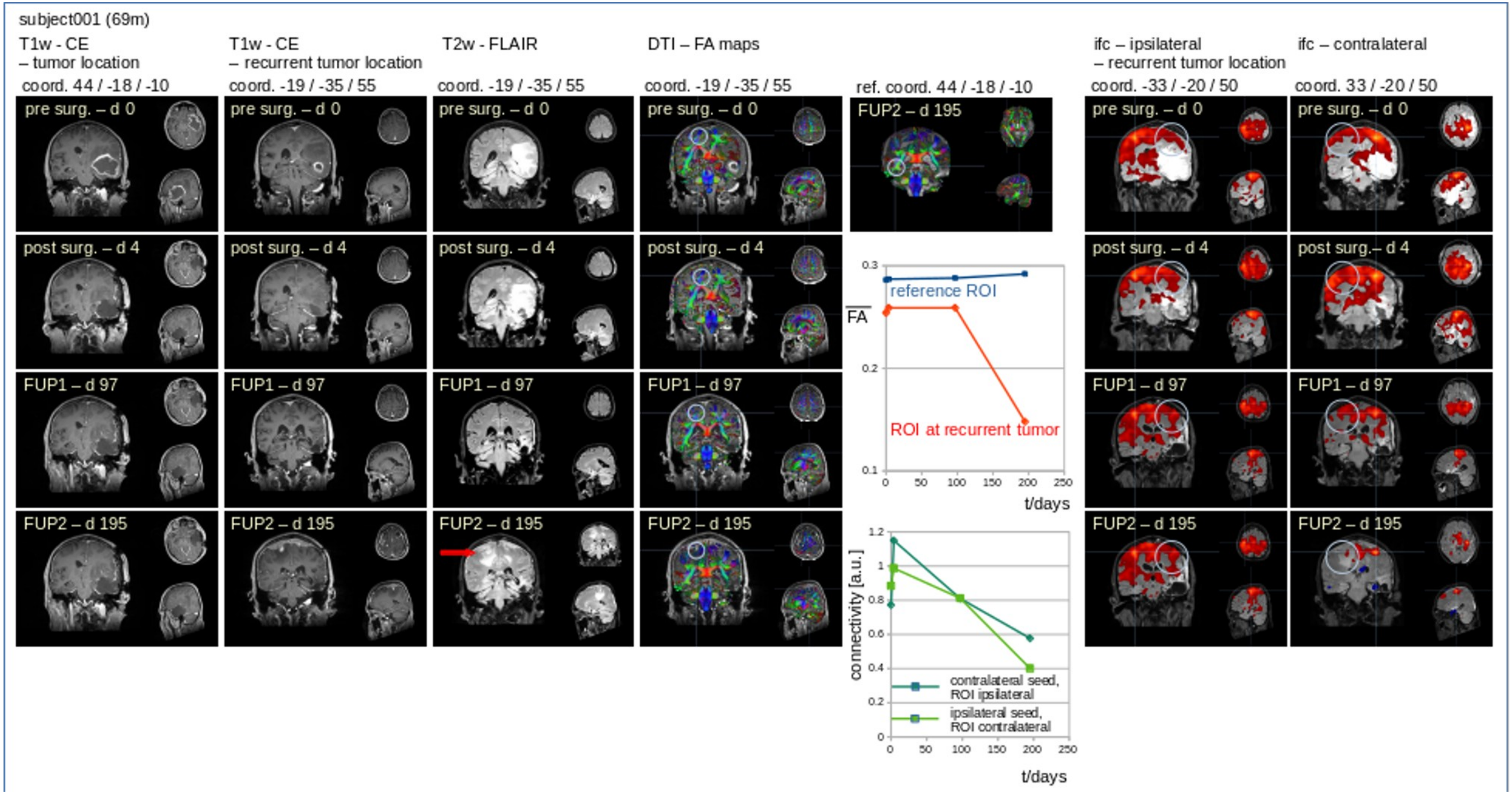

Supplementary Figure 2: Multiparametric MRI analysis for subject 002. Panels from left to right: T1w-contrast enhanced (CE), T2w-FLAIR, FA maps, ICN from rsfMRI. Parameterized longitudinal FA alterations and longitudinal ICN alterations were displayed over time.

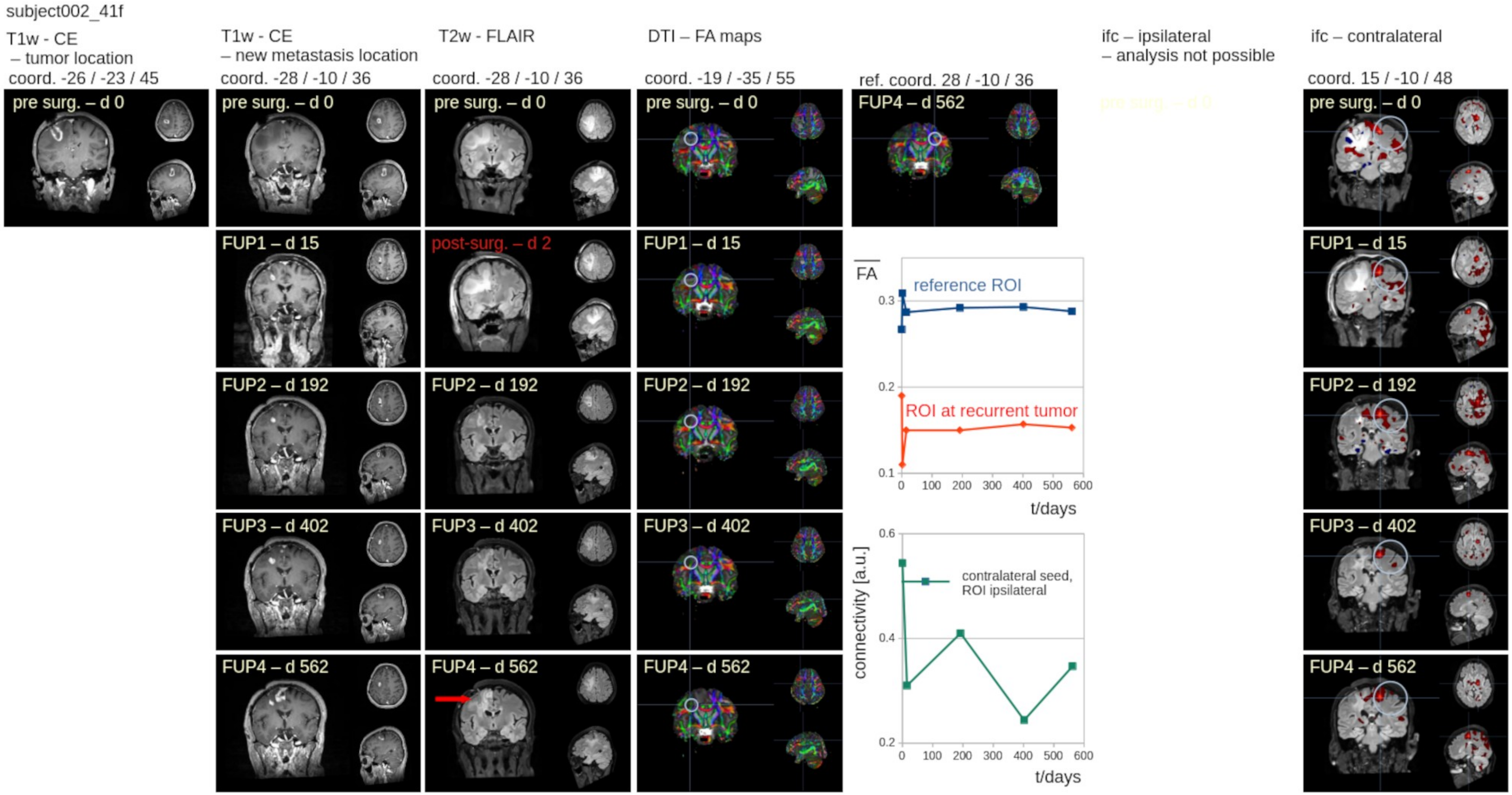

Supplementary Figure 3: Multiparametric MRI analysis for subject 003. Panels from left to right: T1w-contrast enhanced (CE), T2w-FLAIR, FA maps, ICN from rsfMRI. Parameterized longitudinal FA alterations and longitudinal ICN alterations were displayed over time.

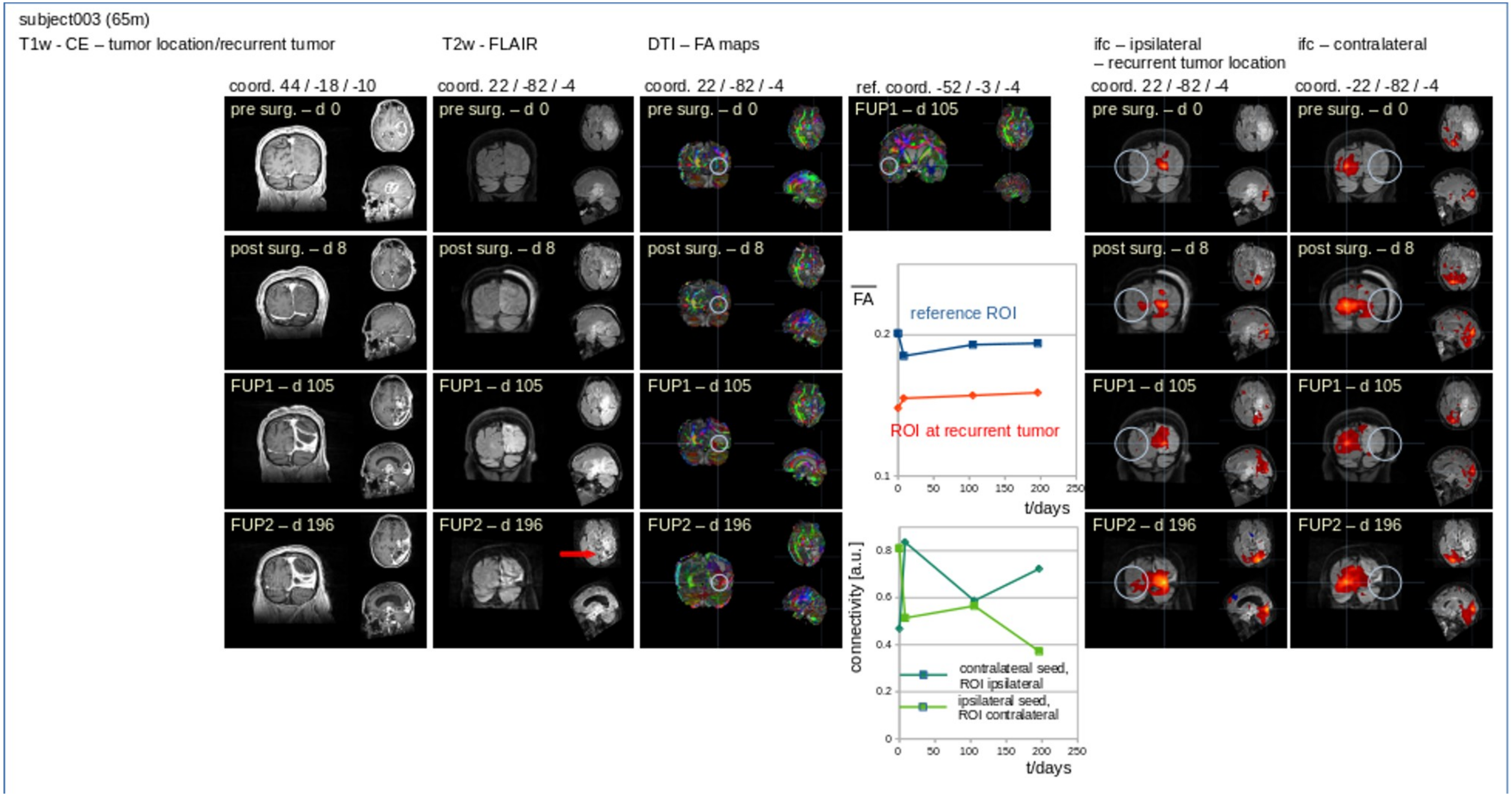

Supplementary Figure 4: Multiparametric MRI analysis for subject 004. Panels from left to right: T1w-contrast enhanced (CE), T2w-FLAIR, FA maps, ICN from rsfMRI. Parameterized longitudinal FA alterations and longitudinal ICN alterations were displayed over time.

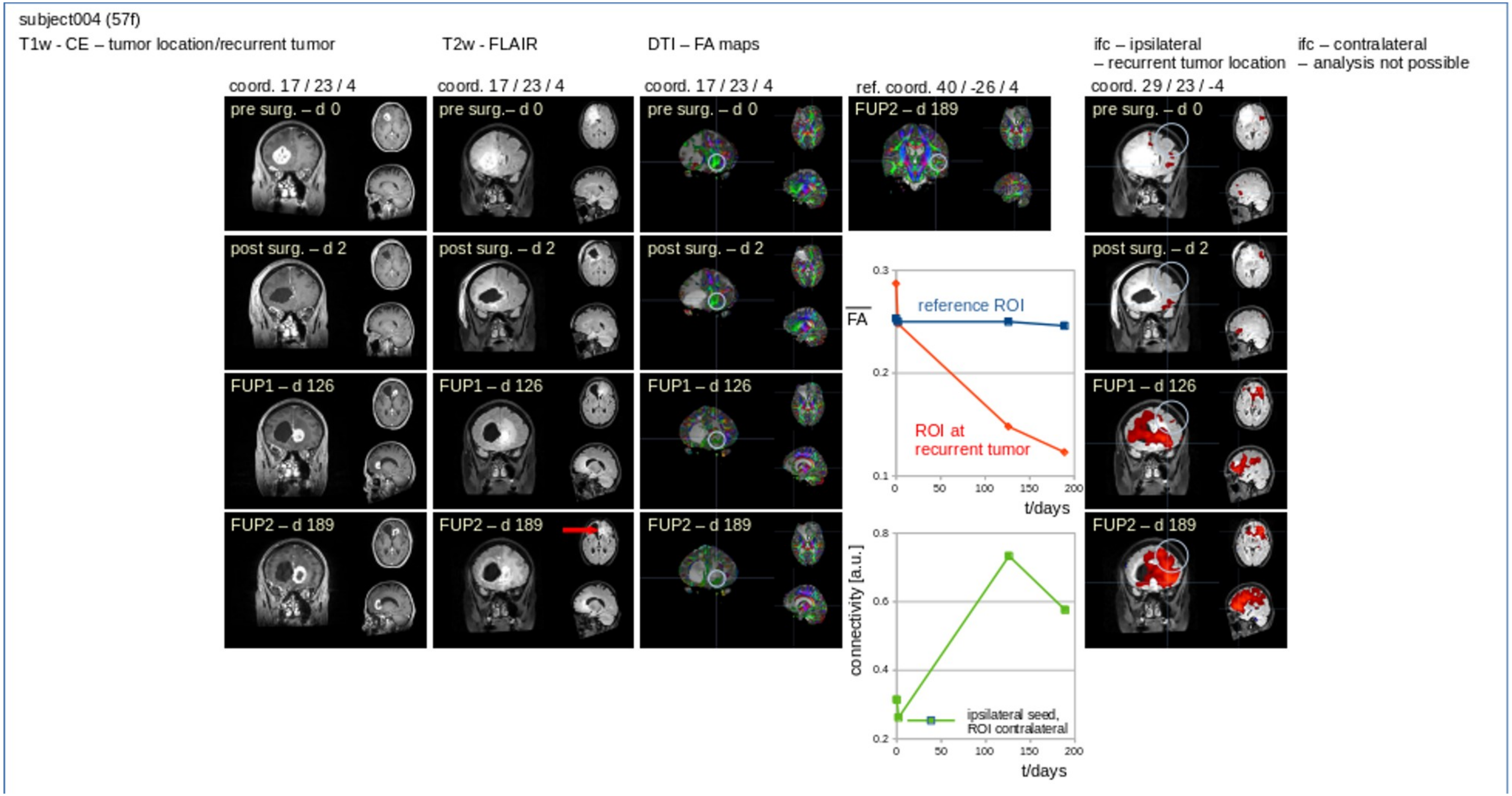

Supplementary Figure 5: Multiparametric MRI analysis for subject 005. Panels from left to right: T1w-contrast enhanced (CE), T2w-FLAIR, FA maps, ICN from rsfMRI. Parameterized longitudinal FA alterations and longitudinal ICN alterations were displayed over time.

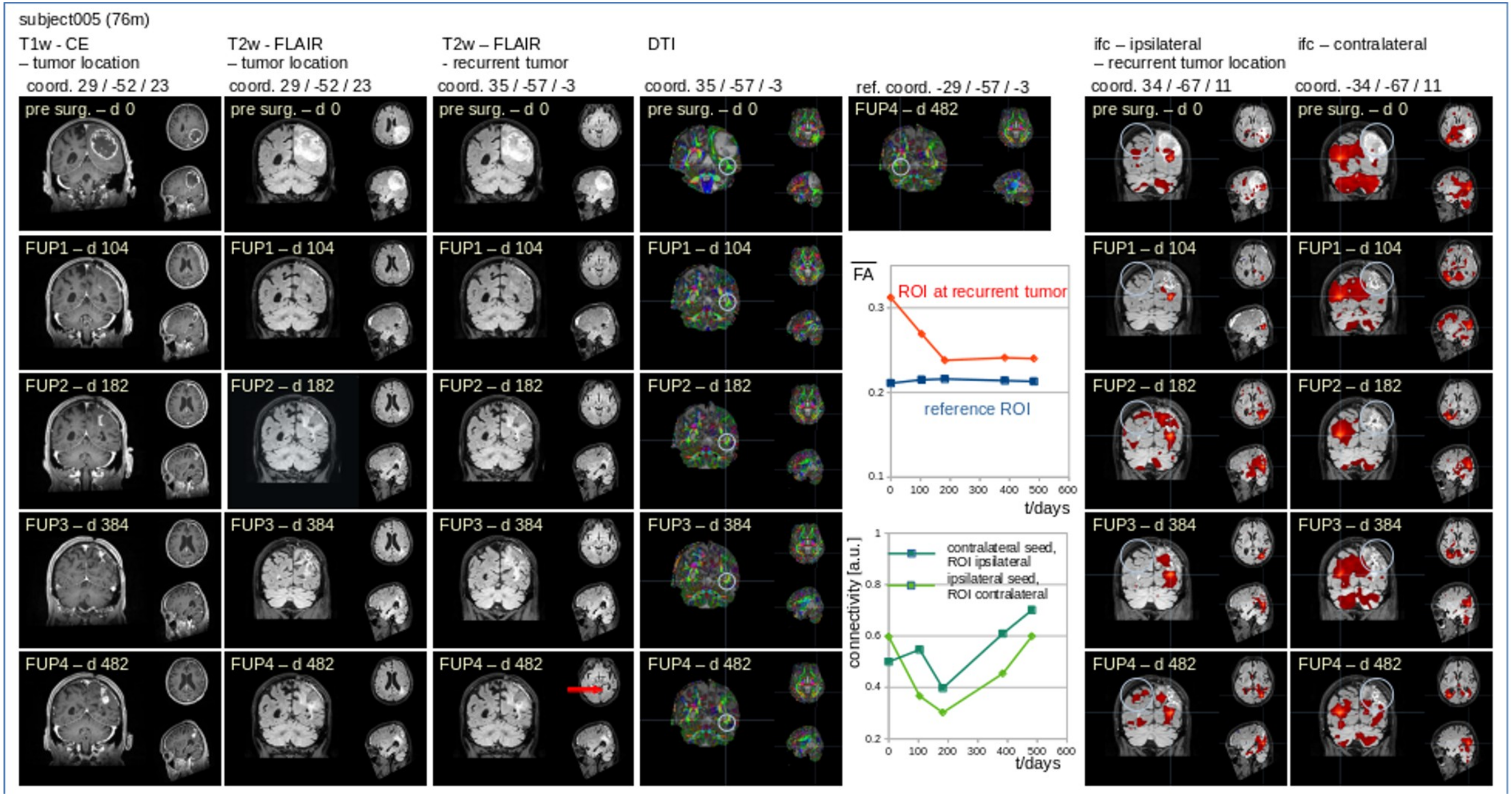

Supplementary Figure 6: Multiparametric MRI analysis for subject 006. Panels from left to right: T1w-contrast enhanced (CE), T2w-FLAIR, FA maps, ICN from rsfMRI. Parameterized longitudinal FA alterations and longitudinal ICN alterations were displayed over time.

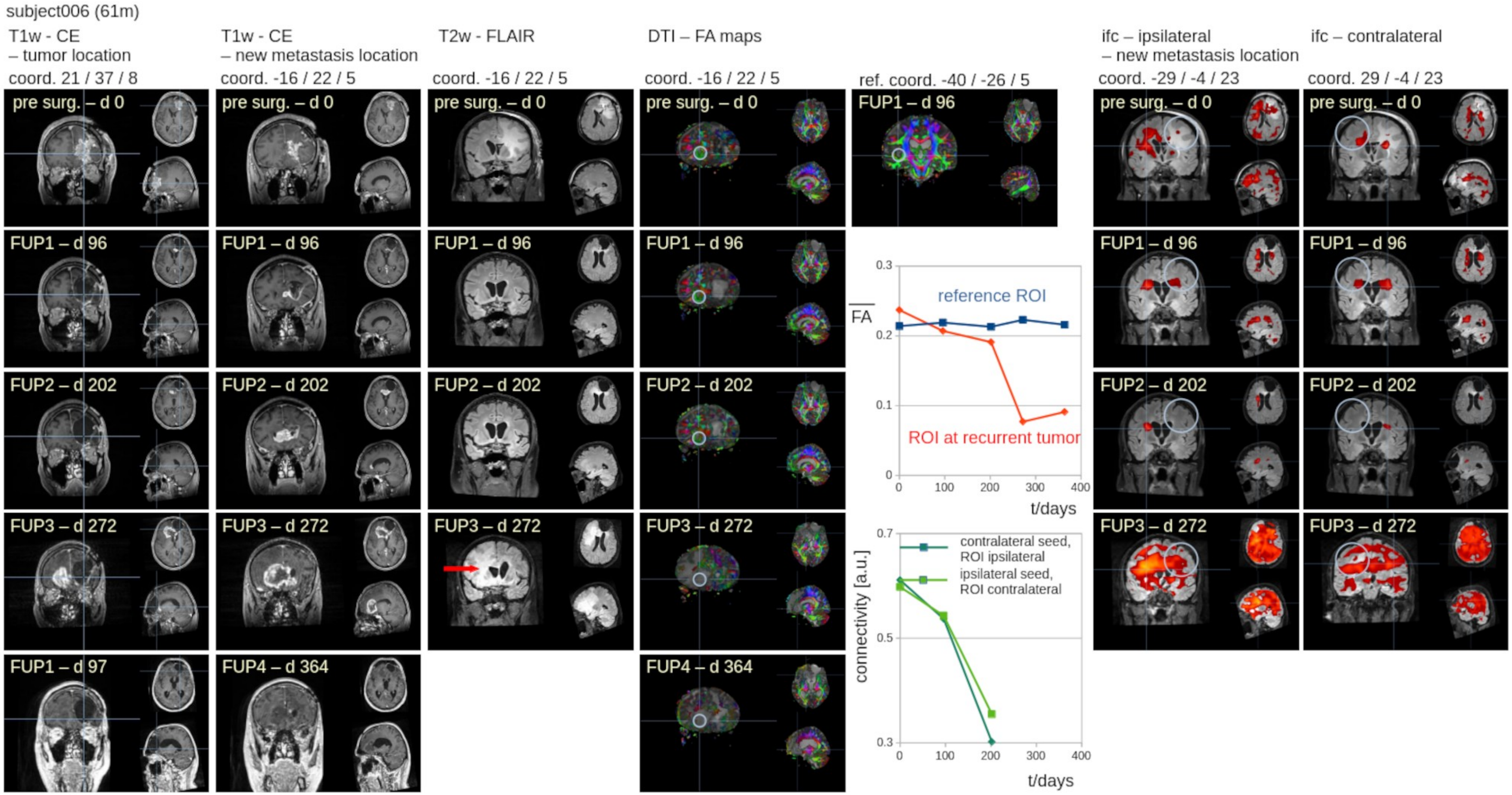

Supplementary Figure 7: Patient's MRI visits in days from first MRI scan (before surgery – t=0). Dropout rate was 9/15. Six patients obtained baseline (pre/post-surgery) and complete MRI (T1w, T2w, DTI, ifcMRI) at a minimum of two follow-up visits.

	pre surgery	post surgery	follow-up 1	follow-up 2	follow-up 3	follow-up 4
subject001	0	4	97	195		
dropout001	0	4	102	no DTI/no IFC	dropout	
subject002	0	2	15	192	402	562
dropout002	0	3	111	dropout		
dropout003	0	2	no DTI/no IFC	no DTI/no IFC	dropout	
dropout004	0	3	99	dropout		
dropout005	0	2	102	no DTI/no IFC	dropout	
subject003	0	8	105	196		
dropout006	0	6	no DTI/no IFC	no DTI/no IFC	dropout	
subject004	0	2	126	189		
dropout007	0	2	no DTI/no IFC	124	dropout	
subject005	0	no DTI/no IFC	104	182	384	482
subject006	0	no DTI/no IFC	96	202	272	364
dropout008	0	7	no DTI/no IFC	dropout		
dropout009	0	4	82	no DTI/no IFC	no DTI/no IFC	dropout
